# Evaluation of Antioxidant Compounds, Antioxidant Activities, and Mineral Composition of 13 Collected Purslane (*Portulaca oleracea* L.) Accessions

**DOI:** 10.1155/2014/296063

**Published:** 2014-01-21

**Authors:** Md. Amirul Alam, Abdul Shukor Juraimi, M. Y. Rafii, Azizah Abdul Hamid, Farzad Aslani, M. M. Hasan, Mohd Asraf Mohd Zainudin, Md. Kamal Uddin

**Affiliations:** ^1^Department of Crop Science, Faculty of Agriculture, Universiti Putra Malaysia (UPM), 43400 Serdang, Selangor, Malaysia; ^2^Institute of Tropical Agriculture, Universiti Putra Malaysia (UPM), 43400 Serdang, Selangor, Malaysia; ^3^Faculty of Food Science and Technology, Universiti Putra Malaysia (UPM), 43400 Serdang, Selangor, Malaysia

## Abstract

The methanolic extracts of 13 accessions of purslane were analyzed for their total phenol content (TPC), total flavonoid contents (TFC), and total carotenoid contents (TCC) and antioxidant activity of extracts was screened using FRAP assay and DPPH radical scavenging methods. The TPC, TFC, and TCC ranged from 0.96 ± 0.04 to 9.12 ± 0.29 mg GAE/g DW, 0.13 ± 0.04 to 1.44 ± 0.08 mg RE/g DW, and 0.52 ± 0.06 to 5.64 ± 0.09 mg (**β**-carotene equivalent) BCE/g DW, respectively. The DPPH scavenging (IC_50_) activity varied between 2.52 ± 0.03 mg/mL and 3.29 ± 0.01 mg/mL and FRAP ranged from 7.39 ± 0.08 to 104.2 ± 6.34 **μ**mol TE/g DW. Among all the measured micro- and macrominerals K content was the highest followed by N, Na, Ca, Mg, P, Fe, Zn, and Mn. The overall findings proved that ornamental purslane was richer in antioxidant properties, whereas common purslane possesses more mineral contents than ornamental ones.

## 1. Introduction

**Figure 1:**
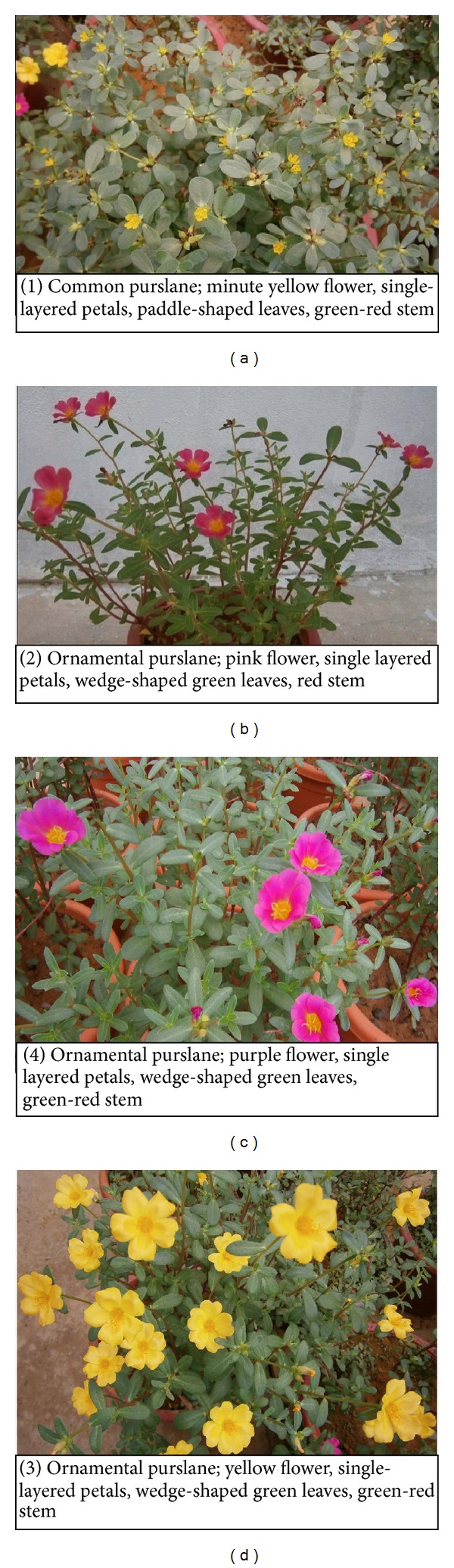
Different types of purslane with brief descriptions.

Antioxidants are vital substances which possess the ability to protect the body from damages caused by free radical-induced oxidative stress, whereas the micro- and macrominerals are the key components for overall body growth and development. Purslane has been ranked as the eighth most common plant in the world [1] and is listed in the World Health Organization as one of the most used medicinal plants and it has been given the term “Global Panacea” [2, 3]. There are about 70 species of edible herbs in Malaysia, which are called by their local name “ulam” [3]. Some of these herbs are claimed to have high antioxidant properties as well as medicinal properties. Purslane is the most prominent candidates among all those herbs. The complex mixture of phytochemicals in vegetables and fruits provides overlapping or complementary effects that contribute to the protective effect of health [4]. The common purslane (Figure 1(a)) locally known as “Gelang Pasir” in Malaysia and Indonesia and ornamental purslane are mainly know as Japanese rose [3]. Purslane is an annual succulent (water content of over 90%), glabrous, prostrate, or ascending plant, 10–70 cm high, very much branched from the base. Leaves are alternate, fleshy, obovate or spathulate with a cuneate base and obtuse apex, smooth and waxy on upper surface, margins are sometimes purple; sessile or indistinctly petiolate, 1–3 cm long, 0.5–1.5 cm wide. Flowers are solitary or clustered axillary or terminal, surrounded by 2 glabrous bracts; 2 unequal sepals, 5 glabrous yellow petals, stamens 6–15. Fruit are brown rounded capsule, 6–10 mm long, opening at top with lid. Seeds are numerous, small, 0.8 mm broad, reniform, and black in color [5–8], whereas ornamental purslane usually does not produce seeds but is potentially propagated by stem cuttings. Leafy vegetables are good source of vitamins and minerals. *Portulaca oleracea* has been reported to be the richest vegetable source of omega-3 (**ω**-3) fatty acids (FA) yet examined [9]. Subsequent reports have confirmed the high levels of **ω**-3 fatty acids and traces of 22: 5**ω**-3 and 22: 6**ω**-3 [10, 11]. Purslane also contains high levels of vitamins E, C, and beta carotene [12]. The abundance of high levels of these essential nutrients in purslane indicates its potential for becoming a new source of nutritious food for both humans and animals. Scientifically, purslane provides a rich plant source of nutritional benefits with high antioxidant properties. It is one of the richest green plant sources of omega-3 fatty acids [9, 10]. In areas where this “weed” is eaten, there is a low incidence of cancer and heart disease, possibly due to purslane's naturally occurring omega-3 fatty acids [12, 13]. Purslane has long been known in Malaysia but still it is underutilized and considered as a weed. To our knowledge, no data on nutritional quality have been published regarding such many different types of collected purslane accessions. Therefore, the objective of this study was to characterize the nutritional components, antioxidant compounds, and antioxidant activities of collected purslane accessions for cultivars evaluation and to select the better cultivars in an attempt to promote their cultivation as a vegetable crop and safely use for human consumption.

## 2. Materials and Methods

### 2.1. Experimental Site and Soil

The experiment was conducted during May 2012 to September 2012 in a glasshouse at the Faculty of Agriculture, University Putra Malaysia (3°00′21.34′′N, 101°42′15.06′′E, 37 m elevation), and Food Biotechnology and Functional Food Research Laboratory, Faculty of Food Science and Technology, UPM, Malaysia. The plastic pots (24 × 22 cm) were filled with soil (39.51% sand, 9.03% silt, and 51.35% clay) of pH 4.8 with 2.6% organic carbon, 1.24 g cc-1 bulk density, and CEC of 7.07 me 100 g^−1^ soil. Soil nutrient status was 0.16% total N, 5.65 ppm available P, 15.3 ppm available K, 3295 ppm Ca, and 321 ppm Mg. At field capacity, soil water retention was 31.18% (wet basis) and 45.31% (dry basis). The experimental soil belongs to the Serdang series.

### 2.2. Plant Materials and Experimental Design

Thirteen (13) samples of 10–15 days young seedlings of purslane were collected from different locations of West Peninsular Malaysia considering location and morphological variation of the plants and transplanted into the pots with prepared soils organized in a randomized complete block design with three replications. Brief descriptions of the collected samples and locations are shown in Table 1 and Figure 1.

### 2.3. Plants Rearing and Sample Collection

Four to five seedlings were transplanted in each pot and were surface-irrigated thrice a week (every alternate day) throughout the growing period using only tape water. Sixty day-old matured harvested purslane samples were divided into two parts of each accession: one for the analysis of antioxidant properties and the other for mineral analysis. Due to light and temperature sensitivity of antioxidant properties the fresh samples were oven-dried at 45–50°C for one week then ground and stored in −20°C freezer until analysis. And for mineral analysis the samples were oven-dried at 70°C for 3 days (72 h) then ground and stored in plastic vials.

### 2.4. Chemicals and Reagents

2,2-Diphenyl-l-picrylhydrazyl solution (DPPH), 2,4,6-tripyridyls-triazine (TPTZ), acetate buffer, ferric chloride (FeCl_3_·H_2_O), sodium acetate hydrate (C_2_H_3_NaO_2_·3H_2_O), aluminum chloride (AlCl_3_·6H_2_O), sodium hydroxide (NaOH), citric acid and 6-hydroxy-2,5,7,8-tetramethylchromane-2-carboxylic acid (Trolox), and Rutin (C_27_H_30_O_16_) were purchased from Sigma-Aldrich Co. (St. Louis, USA); methanol and hexane analytical grade were obtained from HmbG Chemical Co. (Germany); Folin-Ciocalteu reagent and gallic acid were from Merck Co. (Darmstadt, Germany). Sodium carbonate (Na_2_CO_3_) and sodium nitrite (NaNO_2_) were purchased from System Co. (USA). BHA (butylated hydroxyanisole), *α*-tocopherol (C_29_H_50_O_2_), hydrochloric acid (HCl), sulfuric acid (H_2_SO_4_), and hydrogen peroxide (H_2_O_2_) were purchased from Fischer Scientific Co. (Leicestershire, UK).

### 2.5. Sample Preparation and Extraction

Three grams of powdered samples was weighted and placed in 100 mL conical flask and 30 mL of methanol was added with the ratio of 1 : 10 (w/v) and left for 2 hours in water bath shaker with 100 rpm at temperature 40 ± 1°C [14]. The filtrate was separated from the residue by filtering through a filter paper (Whatman number 1) and the residue was reextracted again with fresh solvent according to the procedure mentioned above. The filtrates were pooled and excess methanol was then evaporated off under reduced pressure using a rotatory evaporator (Buchi Rotavapor R-210, Switzerland). The concentrated extract was then stored at −20 ± 1°C prior to analyses. The extracts were prepared by the method described by Crozier et al. [15] with slight modifications.

### 2.6. Determination of Antioxidant Compounds

#### 2.6.1. Determination of Total Phenolic Compounds (TPC)

The TPC were determined using Folin-Ciocalteu method as reported by Singleton et al. [16] with some modifications. 0.5 mL of sample extract at concentration 1 mg/mL was mixed with 0.5 mL Folin-Ciocalteu reagent, followed by addition of 10 mL of 7% sodium carbonate solution. The mixture was allowed to stand for 1 hour at 25 ± 2°C in the dark condition and then absorbance was measured at 725 nm using a UV-Vis Spectrophotometer (UV-1650 PC Spectrophotometer, Shimadzu, Japan). The amount of TPC was expressed as milligram of gallic acid equivalent (GAE) per g of dry weight of sample.

#### 2.6.2. Determination of Total Flavonoid Content (TFC)

Total flavonoid content was determined using a colorimetric method described by Abu Bakar et al. [17]. 0.5 mL of sample extract was mixed with 2.25 mL of distilled water in a test tube followed by addition of 0.15 mL of 5% NaNO_2_ solution. After 6 min, 0.3 mL of a 10% AlCl_3_·6H_2_O solution was added and immediately 1.0 mL of 1 M NaOH was added. The mixture was mixed well by vortex. The absorbance was measured immediately at 510 nm with a spectrophotometer. Results were expressed as milligrams of Rutin equivalent in 1 g of sample (mg RE g^−1^ DW).

#### 2.6.3. Determination of Total Carotenoid Content (TCC)

Total carotenoid content was determined based on the method of Khoo et al. [18] with slight modifications. 0.5 g of dried powered sample was mixed with 15 mL hexane, then vortexed and left for few minutes, and then centrifuged for 1 minute at 3000 rpm. Supernatant was collected and reextracted until it became colorless. Collected supernatant was evaporated until being dried using rotary evaporator at 40°C. Crude extract was redissolved in 5 mL hexane and absorbances were read at 450 nm UV spectrophotometer (UV-1650 PC Spectrophotometer, Shimadzu, Japan). Results were expressed as milligrams of *β*-carotene equivalent in 1 g of sample (mg BCE g^−1^ DW).

### 2.7. Determination of Antioxidant Activity

#### 2.7.1. 1,1-Diphenyl-2-picrylhydrazyl (DPPH) Free Radical Scavenging Activity

Diphenyl-2-picrylhydrazyl (DPPH) assay was used to determine the free radical scavenging activity according to the method of Brand-Williams et al. [19] with some modifications. Sample solutions with different concentrations (0.156 mg/mL to 10 mg/mL) were prepared from purslane extract. An aliquot of each concentration (0.25 mL) was mixed with 1.75 mL of DPPH solution (0.025 mg/mL). The mixture was then left at room temperature for 30 minutes in the dark. Butylated hydroxyanisole (BHA) and *α*-tocopherol at concentrations 100 and 200 ppm were used as a standard, respectively. The absorbance of the mixture was measured at wavelength 515 nm using microplate reader (Model EL-800, BIOTEK Instrument, USA). IC_50_ value, representing the amount of extract which scavenged/reduced 50% of the DPPH radical, was calculated from percent scavenging versus concentration curve. A higher concentration to reduce 50% of DPPH solution showed lower antioxidant activity. The calculation of % DPPH free radical scavenging is as follows:

(1) %  Scavenging  =Absorbance  of  control−Absorbance  of  sample  Absorbance  of  control   ×100%.

#### 2.7.2. Ferric Reducing Antioxidant Power (FRAP) Assay

FRAP assay was conducted based on the method as described by Benzie and Strain [20] with minor modifications. The oxidant in the FRAP assay was prepared by mixing 2.5 mL of 10 mM TPTZ prepared in 40 mM HCl, 25 mL of acetate buffer, and 2.5 mL of 20 mM FeCl_3_·H_2_O. The mixture was referred to as “FRAP reagent.” 200 *μ*L of sample was pipetted into a test tube and mixed with 3 mL of FRAP reagent by vortexing. The mixture was allowed to react for 30 minutes at temperature of 37°C. Absorbance of the mixture was then read at 594 nm. Triplicate tubes were prepared for each extract. The FRAP values expressed in mg Trolox per g were derived from the standard curve.

#### 2.7.3. Chemical Analysis of Purslane Samples

Plant samples were dried in an oven at 70°C for 72 h. Oven-dried samples of purslane were ground and stored in plastic vials until analysis. The N, P, K, Na, Ca, Mg, Fe, Zn, and Mn contents were analyzed using the digestion method [21] and determined using an Atomic Absorption Spectrophotometer (AAS; Perkin Elmer, 5100, USA). For this purpose the grinded powdered samples of 0.25 g were weighed and poured into a digestion tube. Then 5 mL of concentrated sulfuric acid (H_2_SO_4_) was added and kept for overnight or at least 2 hours until the plant materials moistened properly. Then 2 mL of 50% hydrogen peroxide (H_2_O_2_) was added slowly and the digestion tube was placed in a digestion block where the digester block was set to heat for 45 minutes. After 45 minutes the tube was removed and allowed to cool and 2 mL of 50% H_2_O_2_ was added again, kept for heating, and cooling process was repeated until the digested solution became colorless or clear. The cleared cool sample was then filtered and the final volume was made 100 mL by adding distilled water for analysis.

### 2.8. Statistical Analysis

Analysis of variance (ANOVA) procedure in SAS (Version 9.2) [22] was used with a completely randomized design; LSD test was performed to compare the data. All determinations were done at least in triplicates and all were averaged. The confidence limits used in this study were based on 95% (*P* < 0.05).

## 3. Results

### 3.1. Total Phenolic Compounds, Total Flavonoid Contents, and Total Carotenoid Contents

**Figure 2:**
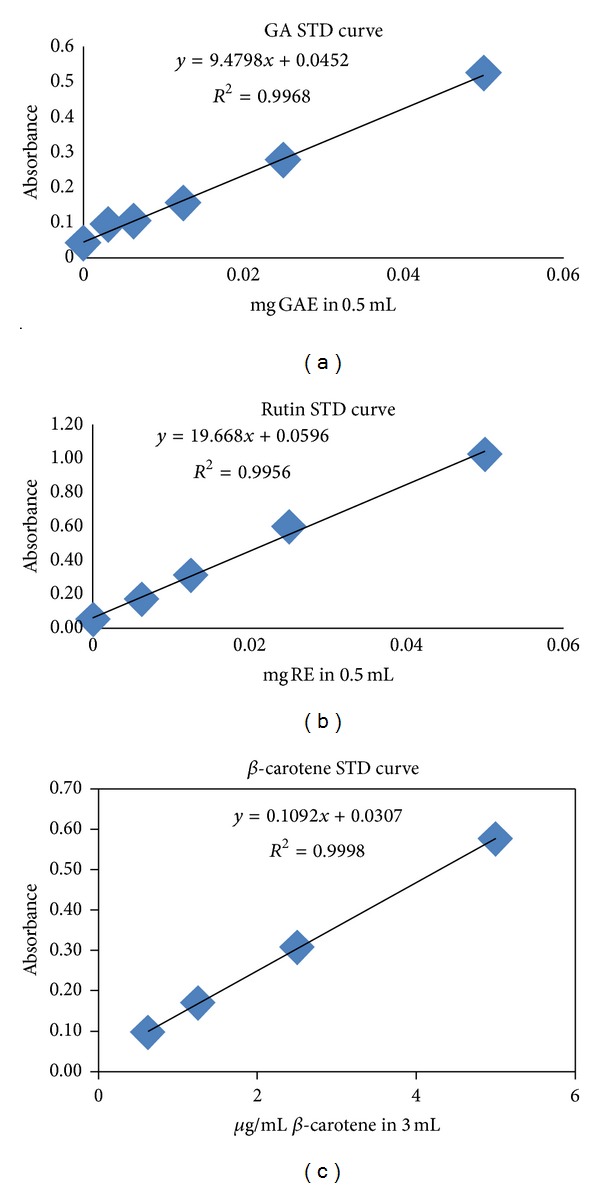
Standard curve of GA (for TPC), Rutin (for TFC), and *β*-carotene (for TCC).

In this study, TPC were determined compared with standard gallic acid, and results are expressed in terms of milligrams of gallic acid equivalent (mg GAE/g dry sample) (Std. curve Figure 2(a)). Total flavonoid content (TFC) was expressed in milligrams of rutin equivalent (mg RE/g dry sample) (Std. curve Figure 2(b)). And total carotenoid content (TFC) was expressed in milligrams of *β*-carotene equivalent (mg BCE/g dry sample) (Std. curve Figure 2(c)).

The TPC, TFC, and TCC values for purslane fraction extracts are presented in Table 2. Among all 13 accessions, the highest amount of TPC (9.12 mg/g DW) was found in accessions V8 and the highest amount of TFC (1.44 mg/g DW) was determined in accessions V10, whereas the highest amount of TCC (5.64 mg/g DW) was found in accessions V4, respectively.

### 3.2. Antioxidant Activity of Purslane

#### 3.2.1. DPPH Free Radical Scavenging Activity

Antioxidant activity of purslane extract that was measured by DPPH assay is presented in Table 3 with the regression equation (*r* ^2^) of BHA and tocopherol standard curve. IC_50_ value for the 13 accessions of purslane extract in the present study was determined and varied between 2.52 ± 0.03 mg/mL and 3.29 ± 0.01 mg/mL indicating the highest antioxidant activity (2.52 mg/mL) shown by the purslane accession V12, whereas the lowest (3.29 mg/mL) was found in accession V9 among all the 13 accessions.

#### 3.2.2. Ferric-Reducing Antioxidant Power (FRAP) Assay

**Figure 3:**
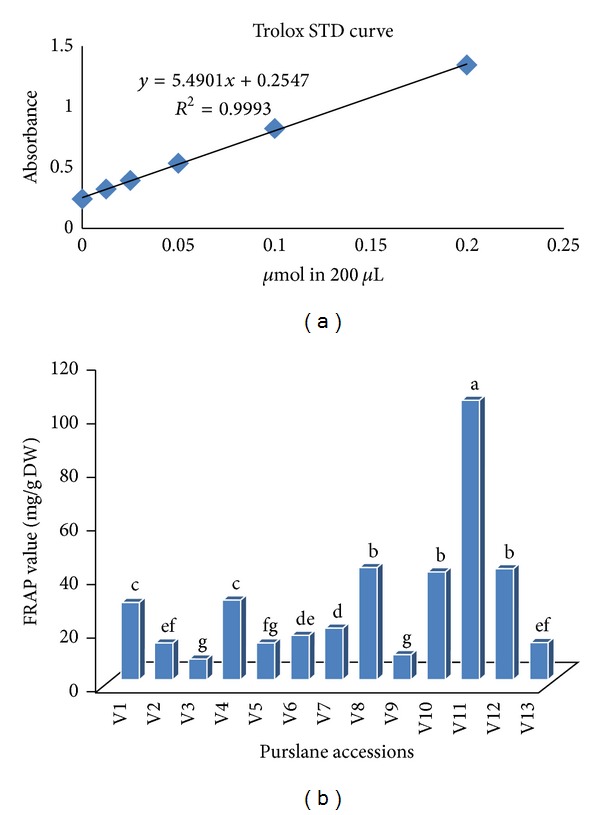
Trolox standard curve with FRAP assayed value. Means followed by the same letter within a column are not significantly different at *P* ≤ 0.05 (LSD).

The ferric-reducing antioxidant power (FRAP) values were measured from all the 13 collected purslane extracts and expressed in mg Trolox per g (Figure 3(a)). There was a significant difference among the FRAP values and they ranged between 104.2 ± 6.34 mg/g DW and 7.39 ± 0.08 mg/g DW as shown in Figure 3(b).

### 3.3. Mineral Composition

Results represented significant variations (*P* < 0.05) in macrominerals (N, P, K, Na, Ca, and Mg) and microminerals (Fe, Zn, and Mn) among all the 13 purslane accessions (Tables 4 and 5).

Maximum concentration of macrominerals N, P, K, Na, Ca, and Mg (184.6 ± 0.84, 14.18 ± 0.22, 656 ± 14, 154.4 ± 0.22, 104.2 ± 0.28, and 101.4 ± 1.46 g/kg DW) was produced by the accessions V12, V13, V11, V11, V6, and V1, respectively, and the minimum of N, P, K, Na, Ca, and Mg (79.3 ± 16.14, 5.08 ± 0.24, 266 ± 12, 52.4 ± 1.9, 35 ± 0.56, and 40.8 ± 0.5) was found in accessions V4, V4, V1, V1, V10, and V2, respectively. Regarding the microelements iron (Fe) content ranged between 11.1 ± 0.18 and 1.86 ± 0.18 g/kg DW (V6 and V7), zinc (Zn) 1.48 ± 0.12 and 0.62 ± 0.06 (V12 and V9), and manganese (Mn) 1.64 ± 0.26 and 0.14 ± 0.04 g/kg DW (V12 and V5 also V7), respectively.

The correlation analysis of the selected macro- and micromineral showed that similar parameter has a highly significant correlation, while among other parameters the correlation is either nonsignificant or less significant or has moderate relation (Table 6).

## 4. Discussion

### 4.1. Total Phenolic Compounds (TPC), Total Flavonoid Contents (TFC), and Total Carotenoid Content (TCC) in Purslane

Phenolic compounds, which are widely distributed in plants [23], have gained much attention because of their antioxidant activities and ability to scavenge free radicals. Antioxidants have potential benefits to human health through their physiological activity, including antioxidant, antimutagenic, and antitumor [23, 24]. Phenolic compounds found in dietary and medicinal plants have shown potential oxidative stress inhibition [25, 26]. Total phenol and total flavonoid content have been reported to be associated with antioxidation activity in various plants [27]. On the other hand, carotenoids are a group of phytochemicals that are responsible for different colors of the foods. They are recognized as playing an important role in the prevention of human diseases and maintaining good health especially against cardiovascular diseases and certain cancers [28, 29]. Based on epidemiological studies a positive link is suggested between higher dietary intake and tissue concentrations of carotenoids and lower risk of chronic diseases [30–32]. The antioxidant properties of carotenoids have been suggested as being the main mechanism by which they afford their beneficial effects. Recent studies are also showing that carotenoids may mediate their effects via other mechanisms such as gap junction communication, cell growth regulation, modulating gene expression, and immune response and as modulators of Phase I and II drug metabolizing enzymes [33–36].

Thirteen collected purslane samples were analyzed in this study for the contents of total phenolic compounds (TPC), total flavonoid contents (TFC), and total carotenoid contents (TCC). Results of the study have been presented in Table 2. From the analysis results, we observed that the TPC contents ranged between 9.12 ± 0.29 mg/g DW and 0.96 ± 0.04 mg/g DW, TFC content 1.44 ± 0.08 mg/g DW and 0.13 ± 0.04 mg/g DW, and TCC 5.64 ± 0.09 mg/g DW and 0.52 ± 0.06 mg/g DW, respectively. Uddin et al. [37] and Lim and Quah [38] also found closely similar results using methanol solvents from matured purslane plants. Among all the 13 accessions V1, V3, V9, and V11 are morphologically more or less the same; on the other hand, V2, V4, V5, and V10 are also morphologically more or less the same but collected from different locations and showed significant (*P* < 0.05) difference regarding TPC, TFC, and TCC contents but Lim and Quah [38] reported that they got near about similar results from the same cultivars collected from different places.

### 4.2. Antioxidant Activity Assay (DPPH and FRAP)

In the present study antioxidant activity was analyzed utilizing 2,2-diphenyl-l-picrylhydrazyl (DPPH) free radical scavenging capacity and ferric ion reducing antioxidant power (FRAP) assay systems and all the findings are shown in Table 3. The IC_50_ is defined as the concentrations required to reduce 50% of DPPH from the original concentration. IC_50_ value for the 13 accessions of purslane extract in the present study was determined and the values were varied between 2.52 ± 0.03 mg/mL and 3.29 ± 0.01 mg/mL indicating the highest antioxidant activity (2.52 mg/mL) shown by the purslane accession V12, whereas the lowest (3.29 mg/mL) was found in accession V9 among all the 13 accessions. This result is partially similar to the findings of Uddin et al. [37] and Siriamornpun and Suttajit [39] at 60 days maturity stage using DPPH assay. Very interestingly we found that the ornamental purslane (V1 to V11) comparatively showed the lower DPPH activity (IC_50_ value) compared with both of the common purslane (V12 and V13) accessions, which represents different trends than TPC, TFC, TCC, and FRAP assays. This finding is same as the results reported by Lim and Quah [38] who worked with both types of purslane. We also found that among all the 13 accessions, V1, V3, V9, and V11 are morphologically more or less the same; on the other hand, V2, V4, V5, and V10 are also morphologically more or less the same but collected from different locations and showed significant (*P* < 0.05) difference both in % inhibition and IC_50_ value. It may happen due to the effect of different geographical locations and microclimatic variations as well as variations in soil qualities.

Antioxidant activity of purslane measured by FRAP assay is presented in Figure 3(b). According to the analysis results it was found that the highest FRAP value (104.2 ± 6.34) was produced by the accession V11 and the lowest (7.39 ± 0.08 mg/g DW) was produced by the accessions V3. This finding is partially similar to the results described by Uddin et al. [37]. Here we observed the same results for V1 and V4; V8, V10, and V12; V3 and V9 and V2 and V13 for FRAP values, though they are not morphologically same but collected from different locations.

### 4.3. Macro- and Micro-Mineral Composition in Purslane

Vegetables are very rich source of essential biochemicals and nutrients such as carbohydrates, carotene, vitamins, calcium, iron, ascorbic acid, and palpable concentrations of trace minerals [40, 41]. In this study significant differences (*P* < 0.05) were observed for major micro- and macronutrients among different clones collected from different locations when compared with one another both in intralocational or even in interlocational. According to the analysis results it was found that potassium (K) content was the highest among all the macro- and microminerals followed by nitrogen (N), sodium (Na), magnesium (Mg), calcium (Ca), iron (Fe), zinc (Zn), and manganese (Mn), respectively. A number of reports have indicated that purslane plants contain the maximum amount of potassium (K) [37, 42, 43].

Because of the reciprocal effects of Na and K authorities have argued that a high diet in potassium and low diet in sodium (low urinary Na and K ratio) favor lower blood pressure. Increase in dietary potassium as the chloride salt has shown to decrease blood pressure in some hypertensive individuals [43]. Low Na and high K diet would decrease the development of cardiovascular disease [44]. Deficiency of calcium, phosphorous, and vitamin D leads to the classic bone symptoms associated with rickets, such as bowlegs, knock knees, curvature of the spine, and pelvic and thoracic deformities [45]. Magnesium plays an important role in the structure and the function of the human body [46].

Iron has the longest and best described history among all the micronutrients. It is a key element in the metabolism of almost all living organisms. In humans, iron is an essential component of hundreds of proteins and enzymes [47, 48]. Manganese (Mn) plays an important role in a number of physiological processes as a constituent of some enzymes and an activator of other enzymes [49]. Zinc plays an important role in the structure of proteins and cell membranes. Loss of zinc from biological membranes increases their susceptibility to oxidative damage and impairs their function [50].

Purslane also contains trace amount of copper (Cu) is an essential trace element for humans and animals [51]. Copper provides the catalytic activity for the antioxidant enzyme copper-zinc superoxide dismutase (Cu, Zn, SOD), while zinc plays a critical structural role [52].

According to the correlation coefficient results, N is negatively correlated with P, Na, and Ca, whereas P is negatively correlated with Ca and Mg, but in case of Mn, it showed significant correlation (*P* < 0.05). K was negatively correlated with Ca and Mg, but Na showed significant negative correlation (*P* < 0.05) with Mg. Ca was negatively correlated with Zn and Mn, whereas Mg was also negatively correlated with Zn, Fe, and Mn. On the other hand Zn showed significant (*P* < 0.05) correlation with Fe, and Fe showed significant correlation with Mn at 0.05% level (Table 6).

## 5. Conclusions

In general, this study demonstrated that different purslane accessions had significantly different bioactive compound content and revealed different levels of responses to biological activity tests. From the overall finding it is found that ornamental purslane has higher TPC (V8), higher TFC (V10), and higher TCC (V4) and antioxidant activities than the common purslane (V12 and V13). Regarding mineral constituents, the common purslane has more mineral composition than ornamental ones. So, we suggest that, both types of purslane are safe for human consumption and very good vegetable food crops for natural minerals, antioxidants, and medicinal purposes.

## Figures and Tables

**Table 1:** Brief descriptions of collected purslane accessions collected from different locations.

Acc. no.	State	Locations	Latitude (°N)	Longitude (°E)	Brief descriptions of the plants
1	Selangor	Tanjung Karang	03°41′	101°19′	Yellow flower, paddle-shaped green leaf, red stem.
2	Selangor	Tanjung Karang	03°41′	101°19′	Pink flower, paddle-shaped green leaf, red stem.
3	Penang	Seberang Perai	05°54′	100°47′	Yellow flower, paddle-shaped, margin green red leaf, red stem leaf
4	Penang	Seberang Perai	05°54′	100°47′	Pink flower, wedge-shaped green red leaf, red stem
5	Kedah	Nursery, Kedah	06°11′	100°37′	Pink flower, wedge-shaped green leaf, red stem
6	Kedah	Nursery, Kedah	06°11′	100°37′	Yellow flower, wedge-shaped green leaf, red stem
7	Kedah	Nursery, Kedah	06°11′	100°37′	Purple flower, paddle-shaped green leaf, red stem
8	Selangor	Sungai Buloh	03°19′	101°59′	White-pink colored flower, wedge-shaped green leaf, red stem
9	Selangor	Sungai Buloh	03°19′	101°59′	Yellow-colored flower, paddle-shaped green leaf, red stem
10	Selangor	Sungai Buloh	03°19′	101°59′	Pink-colored flower, wedge-shaped margin red green leaf, red stem
11	Selangor	AgroBio. UPM	02°98′	101°73′	Yellow-colored flower, red margin wedge-shaped green leaf, red stem
12	N. Sembilan	Kg. Ayer Meleleh	02°54′	101°80′	Wild, yellow flower, paddle-shaped green leaf, red green stem
13	Selangor	Food Sci. UPM	03°01′	101°706′	Wild, yellow-colored flower, wedge-shaped green leaf, green-red stem

**Table 2:** Total phenolic compounds, flavonoid content, and total carotenoid content of 13 accessions of *Portulaca oleracea*.

Accessions	TPC^1^	TFC^2^	TCC^3^
V1	4.28 ± 0.42^c^	0.45 ± 0.03^de^	1.57 ± 0.14^i^
V2	2.64 ± 0.24^gh^	0.55 ± 0.03^d^	2.65 ± 0.11^de^
V3	2.21 ± 2.16^h^	1.18 ± 0.07^b^	3.52 ± 0.04^b^
V4	2.67 ± 0.08^fg^	1.21 ± 0.04^b^	5.64 ± 0.09^a^
V5	0.96 ± 0.04^j^	0.37 ± 0.03^e^	2.05 ± 0.32^gh^
V6	4.1 ± 0.16^cd^	0.37 ± 0.04^e^	1.43 ± 0.16^i^
V7	3.69 ± 0.17^de^	0.89 ± 0.021^c^	2.29 ± 0.11^fg^
V8	9.12 ± 0.29^a^	1.41 ± 0.04^a^	2.41 ± 0.29^ef^
V9	1.64 ± 0.16^i^	0.13 ± 0.04^f^	0.68 ± 0.01^j^
V10	6.58 ± 0.21^b^	1.44 ± 0.08^a^	3.05 ± 0.25^c^
V11	3.38 ± 0.04^f^	0.47 ± 0.01^de^	2.84 ± 0.34^cd^
V12	6.98 ± 0.61^b^	0.94 ± 0.07^c^	1.88 ± 0.05^h^
V13	2.22 ± 0.22^ h^	0.54 ± 0.04^d^	0.52 ± 0.06^j^

^1^mg GAE/g DW. ^2^mg rutin equivalent g^−1^ DW. ^3^mg *β*-carotene equivalent g^−1^ DW.

Means followed by the same letter within a column are not significantly different at *P* ≤ 0.05 (LSD).

**Table 3:** 1,1-Diphenyl-2-picrylhydrazyl (DPPH) radical-scavenging activity of 13 different accessions of purslane.

Purslane accessions	Inhibition ± SD (%)	Regression equation (*r* ^2^)	IC_50_^a,b^ (mg mL^−1^)
V1	59.54 ± 0.39^c^	*y* = 48.72*x* − 94.03 (*r*² = 0.852)	2.95 ± 0.01^c^
V2	48.63 ± 0.33^g^	*y* = 55.89*x* − 94.12 (*r*² = 0.962)	2.58 ± 0.01^h^
V3	66.81 ± 0.56^a^	*y* = 52.89*x* − 100.3 (*r*² = 0.917)	2.83 ± 0.02^e^
V4	60.59 ± 0.67^b^	*y* = 45.68*x* − 83.84 (*r*² = 0.893)	2.92 ± 0.02^d^
V5	41.30 ± 0.38^i^	*y* = 55.73*x* − 100.3 (*r*² = 0.929)	2.70 ± 0.01^f^
V6	53.18 ± 0.25^e^	*y* = 53.36*x* − 114.6 (*r*² = 0.926)	3.09 ± 0.01^b^
V7	50.61 ± 0.29^f^	*y* = 52.89*x* − 84.49 (*r*² = 0.965)	2.54 ± 0.04^ij^
V8	50.66 ± 0.46^f^	*y* = 53.28*x* − 84.59 (*r*² = 0.962)	2.54 ± 0.01^ij^
V9	41.25 ± 0.11^i^	*y* = 58.23*x* − 142.1 (*r*² = 0.950)	3.29 ± 0.01^a^
V10	44.04 ± 0.38^h^	*y* = 59.58*x* − 107.4 (*r*² = 0.937)	2.65 ± 0.01^g^
V11	57.80 ± 0.13^d^	*y* = 47.33*x* − 91.20 (*r*² = 0.857)	2.98 ± 0.00^c^
V12	51.35 ± 1.64^f^	*y* = 58.46*x* − 97.53 (*r*² = 0.936)	2.52 ± 0.03^j^
V13	49.24 ± 0.35^ g^	*y* = 53.97*x* − 87.96 (*r*² = 0.935)	2.56 ± 0.01^hi^

^a^Concentration of sample required to scavenge 50% of free radicals or to prevent lipid peroxidation by 50%.

^b^Means with different letters in the same column are significantly different at *P* < 0.05.

**Table 4:** Selected macromineral composition of 13 collected purslane accessions (on dry weight basis g/kg).

Purslane accessions	N	P	K	Na	Ca	Mg
V1	97.2 ± 11.6^e^*	6.32 ± 0.18^i^	266 ± 12^h^	52.4 ± 1.9^l^	68.4 ± 0.56^f^	101.4 ± 1.46^a^
V2	123.6 ± 8.8^d^	7.34 ± 0.18^g^	304 ± 20^g^	92.4 ± 0.22^d^	48.54 ± 2.3^i^	40.8 ± 0.5^k^
V3	84 ± 8.46^b^	7.48 ± 0.26^g^	462 ± 18^de^	64 ± 0.14^ j^	82 ± 0.5^b^	91.4 ± 0.22^c^
V4	79.3 ± 16.14^b^	5.08 ± 0.24^j^	574 ± 12^c^	57.4 ± 0.28^k^	52 ± 0.26^h^	95.8 ± 0.26^b^
V5	142 ± 0.52^c^	8.44 ± 0.16^d^	502 ± 12^c^	86.6 ± 0.22^f^	64.04 ± 0.56^f^	71.8 ± 0.54^f^
V6	140.3 ± 1.72^c^	8.22 ± 0.26^e^	412 ± 6^f^	85.2 ± 0.34^g^	104.2 ± 0.28^a^	79.6 ± 0.34^d^
V7	136.89 ± 1.72^c^	6.72 ± 0.14^h^	448 ± 12^e^	123 ± 0.18^b^	66.4 ± 0.26^g^	68.8 ± 0.32^g^
V8	183.4 ± 0.92^a^	10.74 ± 0.16^c^	414 ± 12^f^	69.8 ± 0.36^h^	70.4 ± 0.18^e^	68.2 ± 0.42^g^
V9	117.2 ± 1.36^d^	11.4 ± 0.22^b^	502 ± 18^c^	63.8 ± 0.36^j^	75.4 ± 0.44^d^	74.2 ± 0.32^e^
V10	167.2 ± 1.08^b^	6.2 ± 0.18^i^	478 ± 16^d^	66.8 ± 0.26^i^	35 ± 0.56^l^	78.46 ± 2.04^d^
V11	105.24 ± 0.88^e^	7.7 ± 0.14^f^	656 ± 14^a^	154.4 ± 0.22^a^	76.78 ± 0.24^c^	51.62 ± 0.26^h^
V12	184.6 ± 0.84^a^	8.3 ± 0.22^de^	478 ± 14^d^	97.2 ± 0.34^c^	43.2 ± 0.34^j^	46 ± 0.38^j^
V13	118.94 ± 0.64^d^	14.18 ± 0.22^a^	586 ± 16^b^	88.78 ± 0.26^e^	37 ± 0.32^k^	49.6 ± 0.22^i^

Means followed by the same letter within a column are not significantly different at *P* ≤ 0.05 (LSD).

*Average of triplicate determinations ± SD (standard deviation).

**Table 5:** Selected micromineral composition of 13 collected purslane accessions (on dry weight basis g/kg).

Purslane accessions	Fe	Zn	Mn
V1	5.14 ± 0.22^e^	0.86 ± 0.10^cd^	0.34 ± 0.12^gh^
V2	5.26 ± 0.28^e^	0.82 ± 0.04^e^	0.42 ± 0.08^g^
V3	5.72 ± 0.22^d^	0.8 ± 0.08^d–f^	1.06 ± 0.12^c–e^
V4	3.38 ± 0.18^g^	0.7 ± 0.12^e–g^	0.24 ± 0.06^gh^
V5	2.9 ± 0.28^h^	0.98 ± 0.06^bc^	0.14 ± 0.06^h^
V6	11.1 ± 0.18^a^	0.92 ± 0.10^b–d^	0.68 ± 0.14^f^
V7	1.86 ± 0.18^i^	0.66 ± 0.08^fg^	0.14 ± 0.04^h^
V8	6.14 ± 0.26^d^	0.84 ± 0.08^c–e^	1.36 ± 0.22^b^
V9	4.28 ± 0.22^f^	0.62 ± 0.06^g^	1.12 ± 0.18^cd^
V10	3.96 ± 0.30^f^	0.88 ± 0.06^cd^	0.84 ± 0.18^ef^
V11	5.86 ± 0.36^d^	0.78 ± 0.08^d–f^	0.94 ± 0.24^de^
V12	8.66 ± 0.20^c^	1.48 ± 0.12^a^	1.64 ± 0.26^a^
V13	10.06 ± 0.18^b^	1.06 ± 0.10^b^	1.18 ± 0.20^bc^

Means followed by the same letter within a column are not significantly different at *P* ≤ 0.05 (LSD).

*Average of triplicate determinations ± SD (standard deviation).

**Table 6:** Pearson's correlation coefficient among different mineral nutrients of purslane.

Factors	N	P	K	Na	Ca	Mg	Zn	Fe	Mn
N	1								
P	−0.11 ns	1							
K	0.05 ns	0.25 ns	1						
Na	−0.25 ns	0.02 ns	0.42 ns	1					
Ca	−0.17 ns	−0.06 ns	−0.12 ns	0.08 ns	1				
Mg	0.04 ns	−0.43 ns	−0.20	−0.65*	0.36 ns	1			
Zn	0.37 ns	0.20 ns	0.01 ns	0.07 ns	−0.36 ns	−0.43 ns	1		
Fe	0.04 ns	0.49 ns	0.03 ns	0.05 ns	0.14 ns	−0.31 ns	0.57*	1	
Mn	0.39 ns	0.56*	0.24 ns	0.01 ns	−0.12 ns	−0.38 ns	0.49 ns	0.58*	1

Here N, P, K, Na, Ca, Mg, Zn, Fe, and Mn indicate nitrogen, phosphorus, potassium, sodium, calcium, magnesium, zinc, iron, and manganese, respectively. *0.05 > *P* > 0.01; ns: not significant; −: negative correlation at 0.05% level.
